# Percutaneous re-surgical approach for delayed bleeding caused by pseudoaneurysm following percutaneous nephrolithotomy

**DOI:** 10.1016/j.eucr.2023.102551

**Published:** 2023-08-28

**Authors:** Akbar Nouralizadeh, Niloofar Rostaminejad, Negar Radpour, Hamidreza Momeni, Behzad Narouie, Mehdi Dadpour

**Affiliations:** a Department of Urology, Shahid Labbafinejad Medical Center, The Center of Excellence in Urology, Urology and Nephrology Research Center, Shahid Beheshti University of Medical Sciences, Tehran, Iran; b Department of Urology, Zahedan University of Medical Sciences, Zahedan, Iran

**Keywords:** PCNL, Pseudoaneurysm, Hemorrhage

## Abstract

Post percutaneous nephrolithotomy (PCNL) vascular complications included arteriovenous fistula and pseudoaneurysm can cause early or late bleeding and result in unstable condition. Selective *trans*-arterial embolization is the gold standard technique to manage arterial pseudoaneurysm. Herein, we present a case of pseudoaneurysm following PCNL and describe an alternative technique for its removal using a grasper under C-arm vision. Percutaneous re-surgical approach to post-PCNL hemorrhage due to pseudoaneurysm by using a nephro-grasper to pick up the renal artery pseudoaneurysm would be safe, effective and can provide a direct view of pyelocaliceal system for surgeons; And can be a proper alternative for angioembolization.

## 1 Introduction

Percutaneous nephrolithotomy (PCNL) is now the first-line procedure for the treatment of large and complex renal stones.^1^^,^^2^ postoperative complications are including visceral injuries, hemorrhage, arteriovenous fistula and pseudoaneurysm that can be identified through CT or renal angiography.^3^^,^^4^ Pseudoaneurysm is a very rare condition which occurs in 0.6%–1% of the patients. An arterial pseudoaneurysm, also known as a false aneurysm, is caused by damage to the artery wall, resulting in a localized hematoma with turbulent blood flow. These vascular injuries can cause early or late bleeding and result in hemoglobin drop and unstable vital signs for the patient. Selective *trans*-arterial embolization is the gold standard technique to manage arterial pseudoaneurysm following resuscitation.^5^, ^6^, ^7^

Herein, we present a rare event of pseudoaneurysm following PCNL and describe an alternative technique for its removal using a grasper under C-arm vision.

## 2 Case report

**Fig. 1:**
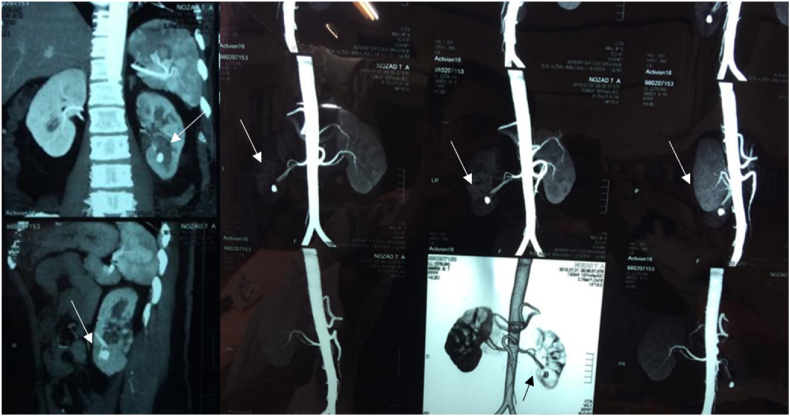
The arrows show pseudoaneurysm in the lower calyx of kidney in different views of CT angiography.

**Case presentation:** A healthy 68-year-old woman with history of PCNL surgery one year ago was referred to our clinic with large kidney stones measuring approximately 20–25 mm in the middle-lower pole of the same side, as observed in the non-contrast CT scan. She underwent PCNL surgery with lower pole access under spinal anesthesia. The procedure lasted approximately 40 minutes without any complications. After two days, both the Foley catheter and the ureteral catheter were removed, and she was discharged from the hospital. Four days later, the nephrostomy tube was removed in the office. After three weeks, she presented to the emergency ward with gross hematuria and unstable vital signs. After patient's resuscitation, a pseudoaneurysm was detected in the middle pole of the kidney at the surgery site by CT angiography (Fig. 1).

**Fig. 2:**
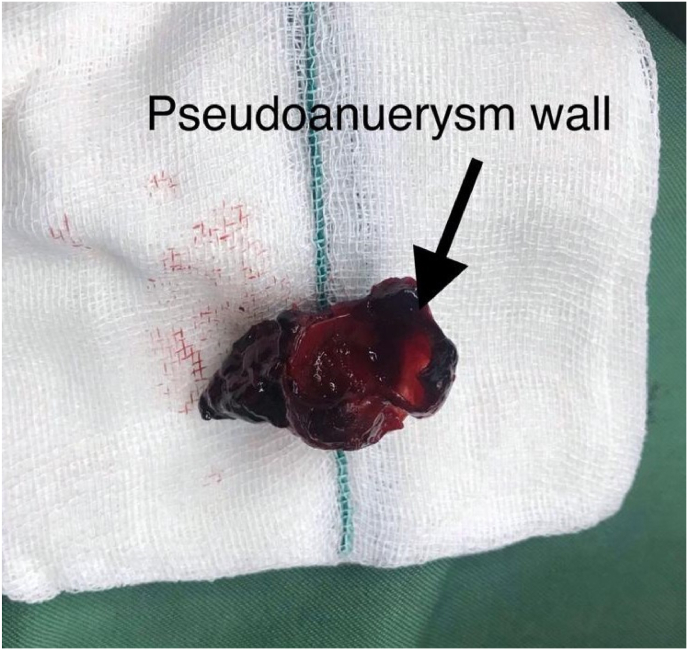
Pseudoaneurysm was removed by a nephro-grasper, under C-arm control.

**Fig. 3:**
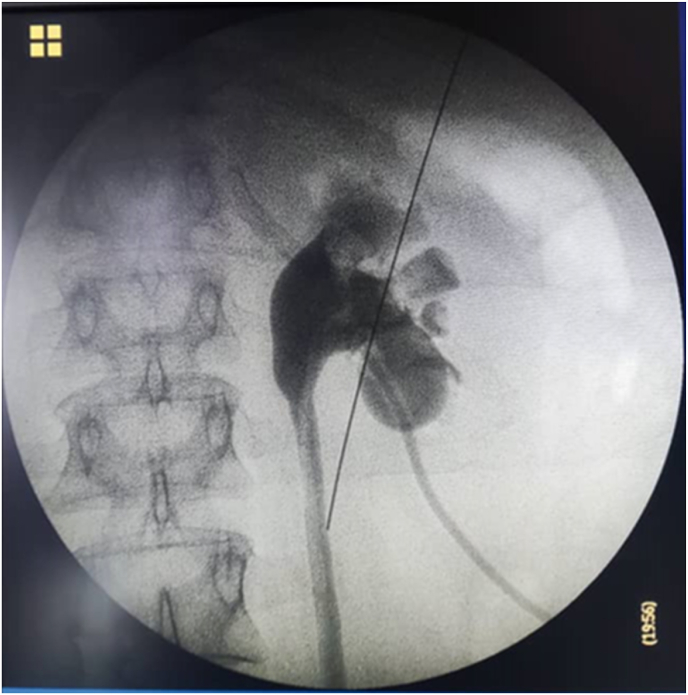
A Foley catheter as a nephrostomy tube was inserted into the lower calyx and the balloon was filled by 4 cc sterile water. Contrast agent from the nephrostomy shows the pyelocaliceal system and the balloon as a filling defect.

**Surgical procedure:** Due to the patient's socio-economic condition and also the unavailability of the angioembolization and its possible high cost, we decided to perform a second PCNL procedure with spinal anesthesia, precisely along the previous nephrostomy line. This allowed us to access the mid-lower pole of the kidney and locate the pseudoaneurysm. After washing the pyelocaliceal system and using a nephro-grasper, under C-arm control, we successfully removed the pseudoaneurysm (Fig. 2), which was found to be filled with floating clot. A Foley catheter as a nephrostomy tube was inserted into the lower calyx and the balloon was filled by 4 cc sterile water (Fig. 3).

**Post-operative care and follow up:** The patient was then admitted to the urology ward for three days, where vital signs and hemoglobin levels were monitored and remained stable. Follow-up evaluation showed no signs of bleeding. After two days, the Foley catheter was removed, and she was discharged from the hospital. Ten days later, the nephrostomy tube was also removed without any complications. A follow-up CT scan after one month revealed no stones or residual pseudoaneurysm. In such cases, after placing the Foley catheter in the calyx and filling the balloon, the balloon will pack the site and stops the bleeding.

## 3 Discussion

Renal artery pseudoaneurysm (RAP) is an unusual complication that may occur very rarely. Beside percutaneous nephrolithotomy, RAPs can also be seen in patients after renal biopsy, partial nephrectomy, or trauma and hematuria is the most common symptom associated with RAP. A high level of clinical suspicion is needed for diagnosis. Noninvasive diagnostic modalities, Eco-color Doppler, CT angiography or digital subtraction angiography are useful diagnostic modalities while Conventional angiography continues to be the standard method for the diagnosis of pseudoaneurysm; It provides endovascular treatment options including embolization.^8^ Despite its advantages, conventional angiography is an invasive procedure that may result in some complications such as development of pseudoaneurysm, arteriovenous fistulas, thrombosis, distal embolization, arterial spasm or ischemia. contrast-enhanced ultrasound is very appropriate tool for monitoring the DSA coil embolization Because of its high sensitivity in detecting low blood flow, and therefore reduces costs and dose exposure in case of RAP. Many reports have shown that coil embolization is an effective method for treating renal pseudoaneurysms following PCNL.^9^

In our patient we used a nephro-grasper to pick up the RAP under fluoroscopy guide and took it out of the kidney. This technique has the potential to reduce the need for angioembolization, which is costly for both the patient and the hospital. Furthermore, performing the procedure under direct surgeon visualization with C-arm control increases the likelihood of success.^10^ Although the gold standard management for such cases is usually angioembolization or open access, our technique appears to be a safe, useful, and cost-effective method for managing pseudoaneurysm following PCNL. However, unpredictable events during re-surgery and lack of experience in management can be its disadvantages.

## 4 Conclusion

Percutaneous re-surgical approach to post-PCNL hemorrhage due to pseudoaneurysm by using a nephro-grasper to pick up the renal artery pseudoaneurysm would be safe, effective, not expensive and can provide a direct view of pyelocaliceal system for the surgeon; And can be a proper alternative for angioembolization.

## Ethics approval and consent to participate

Informed consent from the patients was obtained in this report.

## Consent for publication

The consent was obtained from the patient to publish this report.

## Funding

No fund was used in this project.

## Authors' contributions

AN: project administration, conception, critical review; NR: data curation; NR: drafting, HM: drafting; BN: critical review; MD: drafting, critical review.

## Declaration of competing interest

All authors declare that they have no conflict of interests.

## List of abbreviations

(RAP): Renal artery pseudoaneurysm
(PCNL): Percutaneous nephrolithotomy
